# Supplementary material for: LkARF7 and LkARF19 overexpression promote adventitious root formation in a heterologous poplar model by positively regulating LkBBM1
Source: Commun Biol. 2023 Apr 5;6:372. doi: 10.1038/s42003-023-04731-3 (PMC10076273; doi:10.1038/s42003-023-04731-3)
Supplement: Supplementary file 2 — Supplementary Information [file 42003_2023_4731_MOESM2_ESM.pdf]

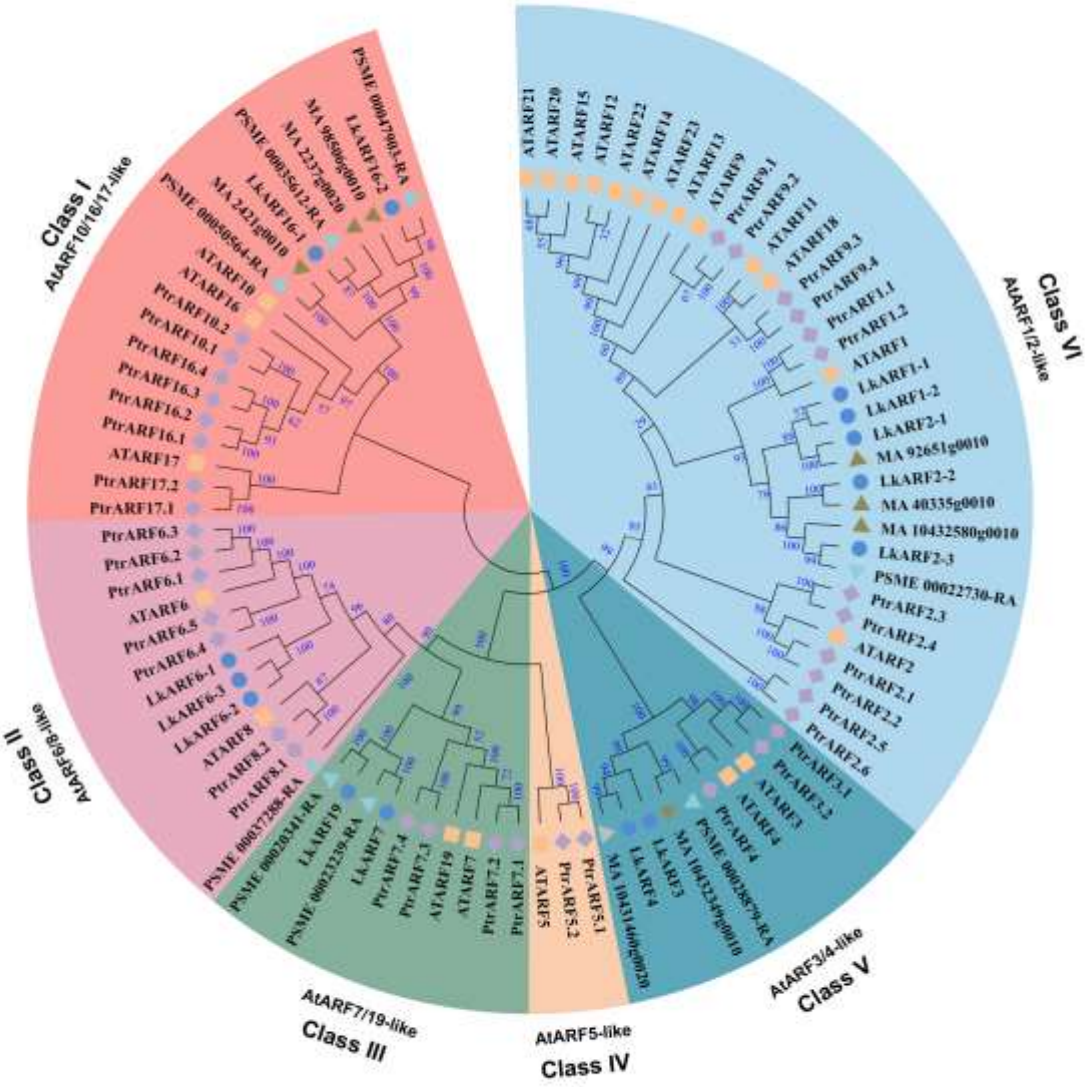

Supplementary Fig. 1. Phylogenetic analysis of ARF proteins in *Larix kaempferi* and other species. ARF amino-acid sequences were aligned using the ClustalW. The phylogenetic tree was constructed with MEGA 7.0 using the neighbor-joining (NJ) method with 1,000 bootstrap replicates. 23 *Arabidopsis thaliana* (AtARF), 36 *Populus trichocarpa* (PtrARF), 14 *L. kaempferi* (LkARF), 8 *Picea abies*, and 8 *Pseudotsuga menziesii* proteins were classified into six subclasses.

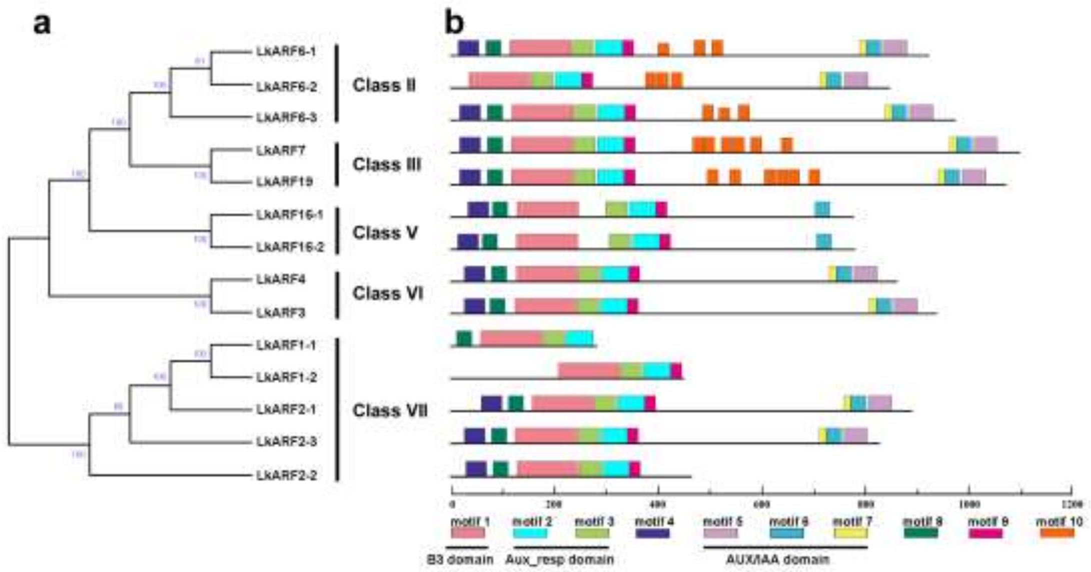

Supplementary Fig. 2. Phylogenetic and motif analysis of LkARFs. **(a)** Phylogenetic analysis of LkARF proteins. The phylogenetic tree was constructed with MEGA 7.0 using the neighbor-joining (NJ) method with 1,000 bootstrap replicates. **(b)** Motif analysis of LkARF proteins. Different motifs are represented by different colors.

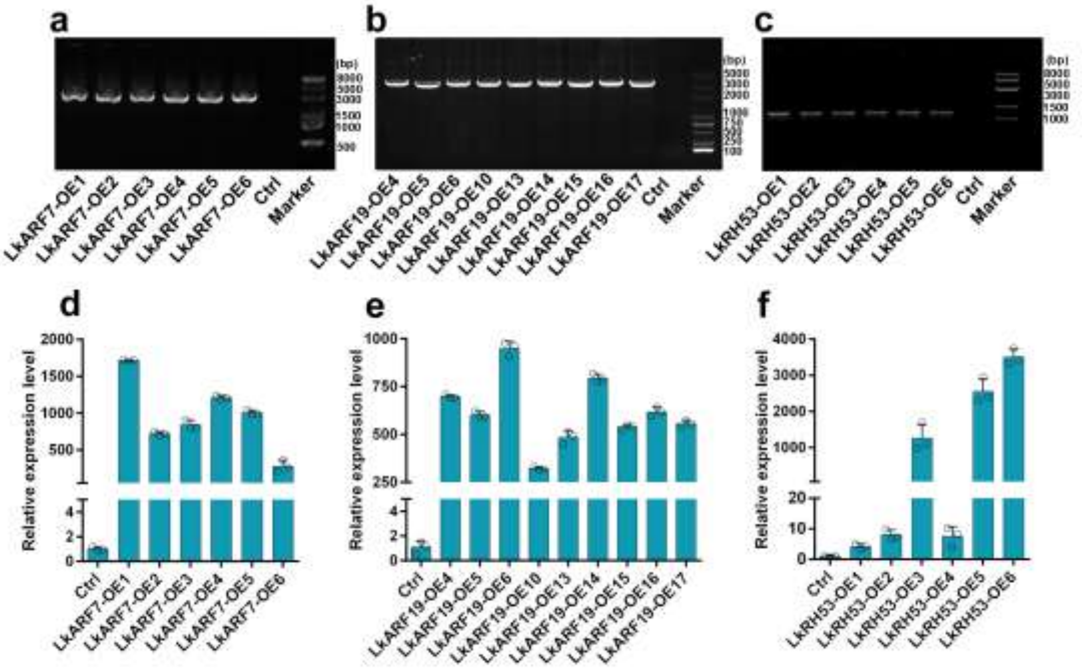

Supplementary Fig. 3. Expression analysis of *LkARF7*-, *LkARF19*- and *LkRH53*-overexpression transgenic poplar lines. Gel electrophoresis identification of **(a)** *LkARF7*-, **(b)** *LkARF19*- and **(c)** *LkRH53*-overexpression transgenic lines by amplification of genomic DNA. qRT-PCR analysis of **(d)** *LkARF7*-, **(e)** *LkARF19*- and **(f)** *LkRH53*-overexpression transgenic lines. *PtoACTIN* was used as the reference gene. Ctrl, non-transgenic 84K poplar. Error bars represent standard deviations from three biological replicates.

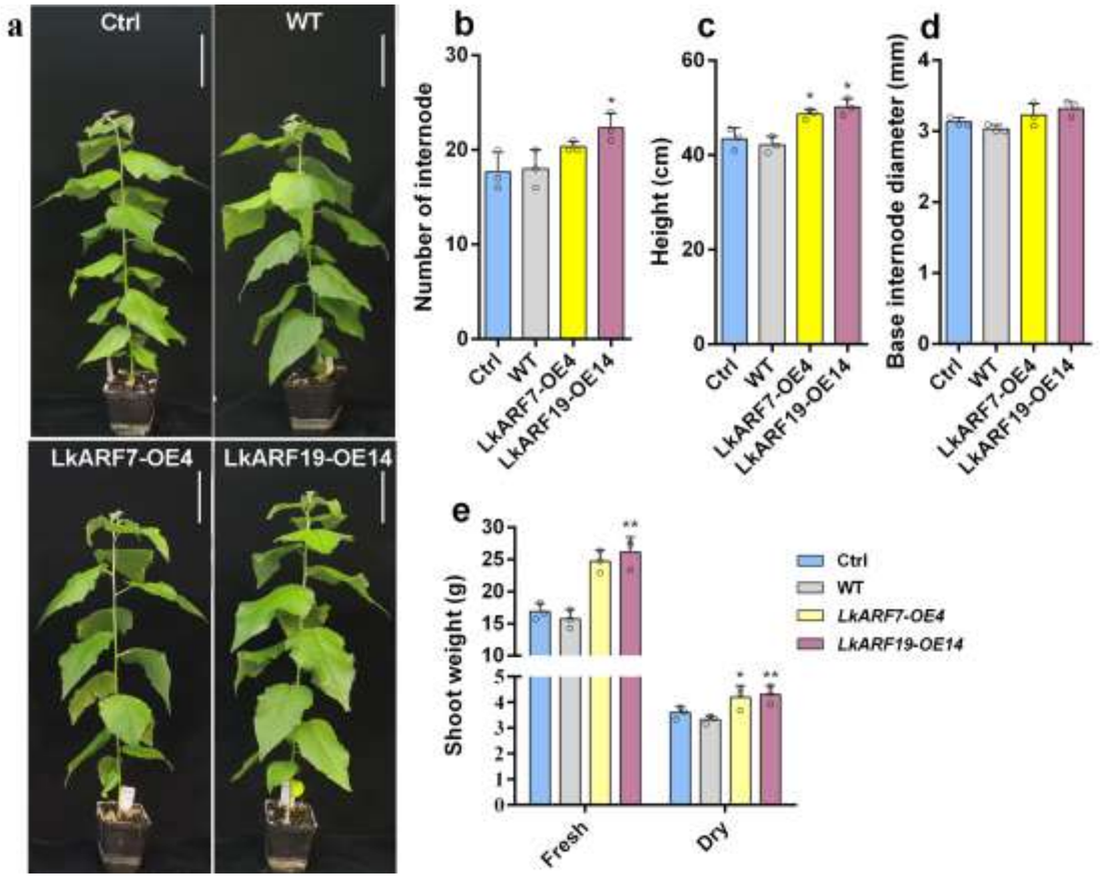

Supplementary Fig. 4. Overexpression of *LkARF7* and *LkARF19* promoted plant growth in transgenic 84K poplar. **(a)** Phenotypes of 8-week-old control, WT and *LkARF7-OE* and *LkARF19-OE* lines. Scale bars, 10 cm. **(b)** number of internode, **(c)** plant height, **(d)** base internode diameter, and **(e)** fresh and dry weight of shoot biomass in control, WT and overexpression lines. Error bars represent standard deviations from multiple biological replicates; *P <0.05 and **P <0.01 *t*-test.

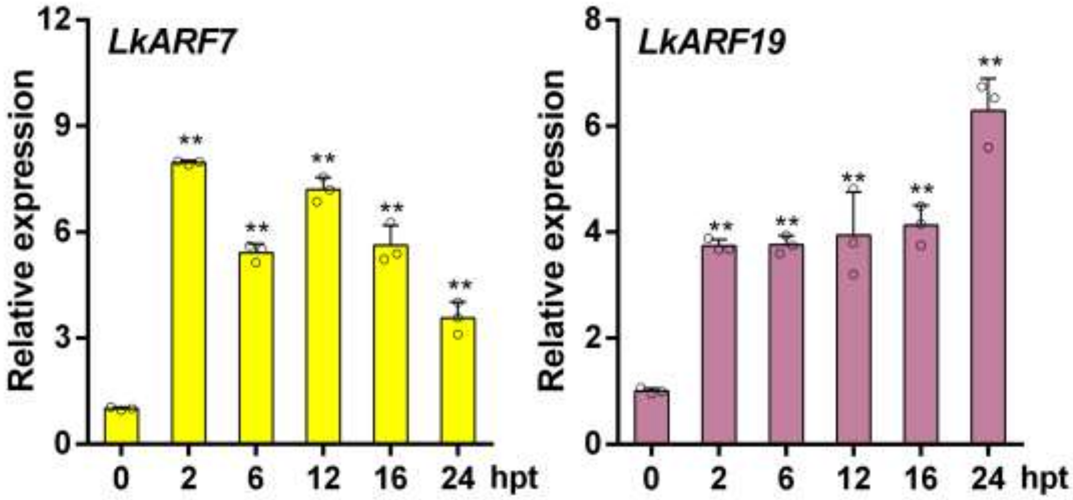

Supplementary Fig. 5. Expression analysis of *LkARF7* and *LkARF19* after exogenous NAA treatment. Relative expression levels of each gene were determined after normalizing to the expression levels in the 0 hpt samples, which were set to 1.0. hpt, hours post treatment. Error bars represent standard deviations from three biological replicates; *P <0.05 and **P <0.01, *t*-test.

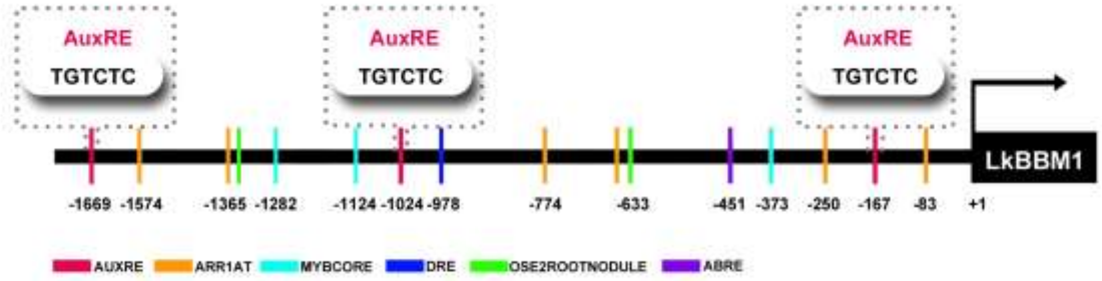

Supplementary Fig. 6. Schematic of *cis*-regulatory elements of the *LkBBM1* promoter. Three auxin response factors elements (AuxREs, TGTCTC) in the promoter of *LkBBM1* are shown. The same colors represent the same elements in different positions. The elements were detected by using the online tool PLACE (http://www.dna.affrc.go.jp/htdocs/PLACE/).

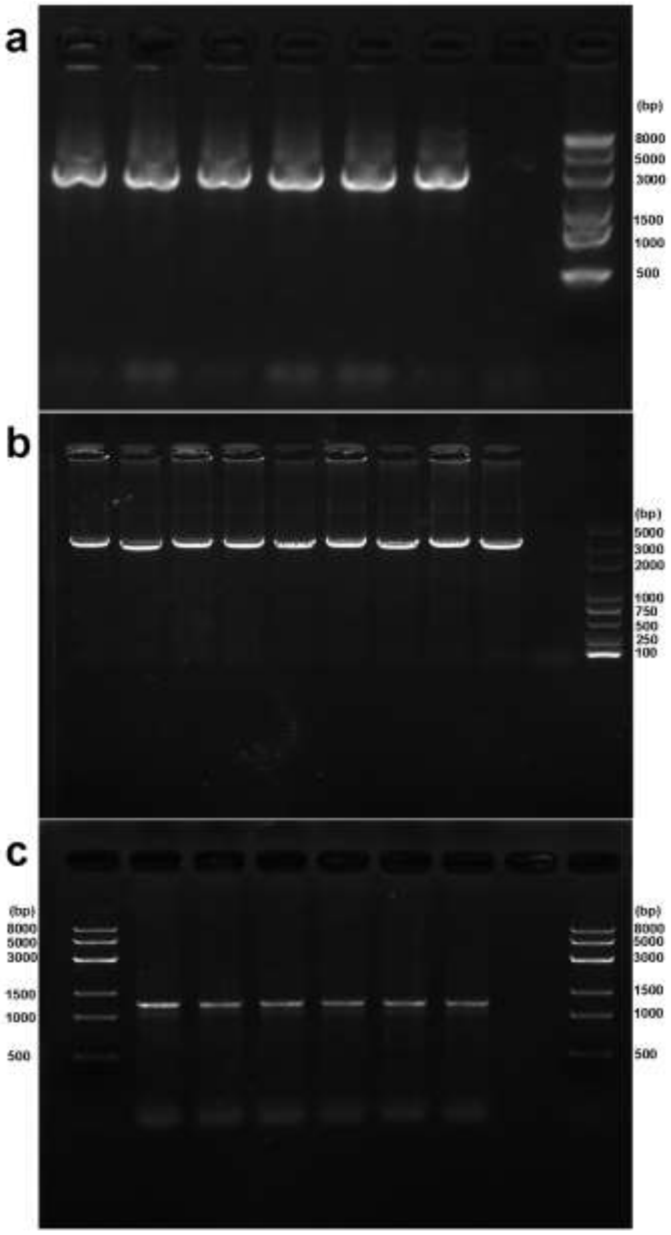

Supplementary Fig. 7. Gel electrophoresis identification of transgenic poplar lines. Electrophoretogram of **(a)** six *LkARF7-OE* lines, **(b)** nine *LkARF19-OE* and **(c)** six *LkRH53-OE* lines, and non-transgenic 84K poplar as shown on Supplementary Fig. 3a – 3c.
