# Supplementary material for: LkARF7 and LkARF19 overexpression promote adventitious root formation in a heterologous poplar model by positively regulating LkBBM1
Source: Commun Biol. 2023 Apr 5;6:372. doi: 10.1038/s42003-023-04731-3 (PMC10076273; doi:10.1038/s42003-023-04731-3)
Supplement: Supplementary file 3 — Description of Additional Supplementary Files [file 42003_2023_4731_MOESM3_ESM.pdf]

Description of Additional Supplementary Files

File name: Supplementary Data 1

Description: Basic information of the *ARF* gene family in *L. kaempferi*.

File name: Supplementary Data 2

Description: The *cis*-acting elements of the *LkBBM1* promoter.

File name: Supplementary Data 3

Description: Specific primers used in the study.

File name: Supplementary Data 4

Description: Description: The source data behind the graphs in the paper
