# Supplementary material for: LkARF7 and LkARF19 overexpression promote adventitious root formation in a heterologous poplar model by positively regulating LkBBM1
Source: Commun Biol. 2023 Apr 5;6:372. doi: 10.1038/s42003-023-04731-3 (PMC10076273; doi:10.1038/s42003-023-04731-3)
Supplement: Supplementary file 8 — Reporting Summary [file 42003_2023_4731_MOESM8_ESM.pdf]

nature portfolio

Corresponding author(s): Xiao-Mei Sun
Last updated by author(s): Mar 8, 2023

Reporting Summary

Nature Portfolio wishes to improve the reproducibility of the work that we publish. This form provides structure for consistency and transparency in reporting. For further information on Nature Portfolio policies, see our Editorial Policies and the Editorial Policy Checklist.

Statistics

For all statistical analyses, confirm that the following items are present in the figure legend, table legend, main text, or Methods section.

n/a	Confirmed	
☐	☒	The exact sample size (*n*) for each experimental group/condition, given as a discrete number and unit of measurement
☐	☒	A statement on whether measurements were taken from distinct samples or whether the same sample was measured repeatedly
☒	☐	The statistical test(s) used AND whether they are one- or two-sided<br>*Only common tests should be described solely by name; describe more complex techniques in the Methods section.*
☒	☐	A description of all covariates tested
☐	☒	A description of any assumptions or corrections, such as tests of normality and adjustment for multiple comparisons
☐	☒	A full description of the statistical parameters including central tendency (e.g. means) or other basic estimates (e.g. regression coefficient) AND variation (e.g. standard deviation) or associated estimates of uncertainty (e.g. confidence intervals)
☐	☒	For null hypothesis testing, the test statistic (e.g. *F*, *t*, *r*) with confidence intervals, effect sizes, degrees of freedom and *P* value noted<br>*Give P values as exact values whenever suitable.*
☒	☐	For Bayesian analysis, information on the choice of priors and Markov chain Monte Carlo settings
☒	☐	For hierarchical and complex designs, identification of the appropriate level for tests and full reporting of outcomes
☒	☐	Estimates of effect sizes (e.g. Cohen's *d*, Pearson's *r*), indicating how they were calculated

Our web collection on statistics for biologists contains articles on many of the points above.

Software and code

Policy information about availability of computer code

Data collection	no software was used
Data analysis	no software was used

For manuscripts utilizing custom algorithms or software that are central to the research but not yet described in published literature, software must be made available to editors and reviewers. We strongly encourage code deposition in a community repository (e.g. GitHub). See the Nature Portfolio guidelines for submitting code & software for further information.

Data

Policy information about availability of data

All manuscripts must include a data availability statement. This statement should provide the following information, where applicable:

- Accession codes, unique identifiers, or web links for publicly available datasets
- A description of any restrictions on data availability
- For clinical datasets or third party data, please ensure that the statement adheres to our policy

The sequences of LkARF7, LkARF19 and LkRH53 have been deposited in NCBI with GenBank accession numbers ON529550, ON529549 and ON529551, respectively.

Human research participants

Policy information about studies involving human research participants and Sex and Gender in Research.

Reporting on sex and gender	N/A
Population characteristics	N/A
Recruitment	N/A
Ethics oversight	N/A

Note that full information on the approval of the study protocol must also be provided in the manuscript.

Field-specific reporting

Please select the one below that is the best fit for your research. If you are not sure, read the appropriate sections before making your selection.

☒ Life sciences ☐ Behavioural & social sciences ☐ Ecological, evolutionary & environmental sciences

For a reference copy of the document with all sections, see nature.com/documents/nr-reporting-summary-flat.pdf

Life sciences study design

All studies must disclose on these points even when the disclosure is negative.

Sample size	Because the sample size was less than 10, we used Student's paired t-test to assess difference.
Data exclusions	No data were excluded.
Replication	For all measurements, we performed at least three biological replications. It was confirmed that we succeeded.
Randomization	We randomly selected samples for analyzing expression of gene and promoter fragments , the relative LUC/REN of firefly luciferase activity, and phenotypes of over-expression plants.
Blinding	There was no group allocation involved in our study during data analysis.

Reporting for specific materials, systems and methods

We require information from authors about some types of materials, experimental systems and methods used in many studies. Here, indicate whether each material, system or method listed is relevant to your study. If you are not sure if a list item applies to your research, read the appropriate section before selecting a response.

Materials & experimental systems

n/a	Involved in the study	
☐	☒	Antibodies
☒	☐	Eukaryotic cell lines
☒	☐	Palaeontology and archaeology
☒	☐	Animals and other organisms
☒	☐	Clinical data
☒	☐	Dual use research of concern

Methods

n/a	Involved in the study	
☒	☐	ChIP-seq
☒	☐	Flow cytometry
☒	☐	MRI-based neuroimaging

Antibodies

Antibodies used	supplier name: CWBIO, catalog number: CW0287M, clone name: Anti Flag-Tag Mouse Monoclonal Antibody, lot number: 01222/18121
Validation	This antibody was applied to Arabidopsis and popular, with references Zhong, S.-H. et al. Warm temperatures induce transgenerational epigenetic release of RNA silencing by inhibiting siRNA biogenesis in Arabidopsis. Proc. Natl. Acad. Sci. U. S. A. 110, 9171–9176 (2013) and He, H. et al. The role of senescence-associated gene101 (PagSAG101a) in the regulation of secondary xylem formation in poplar. J. Integr. Plant Biol. 64, 73–86 (2022).
